# NFE2L2 and STAT3 Converge on Common Targets to Promote Survival of Primary Lymphoma Cells

**DOI:** 10.3390/ijms241411598

**Published:** 2023-07-18

**Authors:** Andrea Arena, Michele Di Crosta, Roberta Gonnella, Roberta Zarrella, Maria Anele Romeo, Rossella Benedetti, Maria Saveria Gilardini Montani, Roberta Santarelli, Gabriella D’Orazi, Mara Cirone

**Affiliations:** 1 Department of Experimental Medicine, Sapienza University of Rome, Viale Regina Elena 324, 00161 Rome, Italy; a.arena@uniroma1.it (A.A.); mariaanele.romeo@uniroma1.it (M.A.R.); rossella.benedetti@uniroma1.it (R.B.); mariasaveria.gilardinimontani@uniroma1.it (M.S.G.M.);; 2 Department of Neurosciences, Imaging and Clinical Sciences, University “G. D’Annunzio”, 66013 Chieti, Italy; gdorazi@unich.it

**Keywords:** PEL, STAT3, NRF2, HSPs, DDR, p62/SQSTM1, autophagy, c-Myc

## Abstract

NFE2L2 and STAT3 are key pro-survival molecules, and thus, their targeting may represent a promising anti-cancer strategy. In this study, we found that a positive feedback loop occurred between them and provided evidence that their concomitant inhibition efficiently impaired the survival of PEL cells, a rare, aggressive B cell lymphoma associated with the gammaherpesvirus KSHV and often also EBV. At the molecular level, we found that NFE2L2 and STAT3 converged in the regulation of several pro-survival molecules and in the activation of processes essential for the adaption of lymphoma cells to stress. Among those, STAT3 and NFE2L2 promoted the activation of pathways such as MAPK3/1 and MTOR that positively regulate protein synthesis, sustained the antioxidant response, expression of molecules such as MYC, BIRC5, CCND1, and HSP, and allowed DDR execution. The findings of this study suggest that the concomitant inhibition of NFE2L2 and STAT3 may be considered a therapeutic option for the treatment of this lymphoma that poorly responds to chemotherapies.

## 1. Introduction

Cancer cells are exposed to stressful conditions, deriving from internal and external insults. KEAP1/NFE2L2 (Kelch-like ECH-associated protein 1-nuclear factor (erythroid-derived 2)-like 2) pathway and heat shock proteins (HSPs) are essential for adaption to stress, not limited to oxidative or thermal stress, to which they mainly respond. NFE2L2, indeed, besides controlling the transcription of enzymes such as superoxide dismutase (SOD), catalase (CAT), NAD(P)H quinone dehydrogenase 1 (NQO1), involved in reactive oxygen species (ROS) detoxification [1], can cooperate with other transcription factors such as heat shock transcription factor 1 (HSF1) to sustain cell survival [2,3]. Indeed, although HSF1 represents the main transcription factor regulating the HSPs, NFE2L2 also positively controls the expression of these proteins [2]. HSPs, classified based on their molecular weight, are usually up-regulated in cancer cells, which therefore become strongly dependent on their expression. As a consequence, HSPs may be considered an Achilles heel whose targeting may allow them to specifically target cancer cells [4] while sparing the normal ones [5]. HSP expression is also under the control of the signal transducer and activator of transcription 3 (STAT3) [6] that may be maintained in an activated state by the HSPs that stabilize the STAT3-phosphorylating kinases [4]. We have recently reported that a positive feedback loop between STAT3 and HSPs also occurs in primary effusion lymphoma (PEL) cells [7], a rare B cell lymphoma associated with Kaposi sarcoma herpesvirus (KSHV) and, in most of the cases, also with Epstein–Barr virus (EBV) [8]. PEL displays a poor response to chemotherapies, and therefore, the search for more effective treatments is urgently needed. No mutations are usually detected in this lymphoma, and therefore, these oncoviruses play a key role in the activation of molecular pathways driving its survival/proliferation. Among those, STAT3 is constitutively phosphorylated in PEL cells either in the tyrosine and serine residues [9], due not only to the expression of viral proteins but also to the release of cytokines, either of cellular and viral origin such as interleukin 6 (IL6), with which STAT3 engages a cross-talk [10]. Among other molecules, STAT3 positively regulates the expression of MYC, survivin (BIRC5), and cyclin D1 (CCND1) [11]. We have recently discussed that STAT3 may interact with NFE2L2, and their interaction may result in a synergic or antagonistic effect with respect to cancer cell survival [12]. In this study, we evaluated whether an NFE2L2/STAT3 cross-talk could occur in PEL cells and investigated the impact of their interplay on cell survival and the molecules involved. Previous studies have reported that STAT3 activation put a brake on autophagy [13], leading to the accumulation of p62/SQSTM1 and NFE2L2 stabilization [14]. Interestingly NFE2L2 may, in turn, promote p62/SQSTM1 transcription in a positive regulatory circuit that has important implications in carcinogenesis [15]. In line with this evidence, we have previously shown that STAT3 inhibition by tyrphostin (AG490) promoted the autophagic flux in PEL cells and reduced the expression level of p62/SQSTM1 [16], being this protein mainly degraded through this catabolic route [17]. Here, besides investigating the interplay between STAT3 and NFE2L2, we evaluated whether the concomitant inhibition of these transcription factors could affect the activation of pathways such as the mammalian target of rapamycin (MTOR) and the extracellular signal-regulated protein kinase 1/2 (MAPK3/1), converge in controlling the expression of pro-survival molecules considered to be NFE2L2 or STAT3 targets and affect the expression of p62/SQSTM1, HSPs, and DNA damage response (DDR) molecules, considered among the HSPs clients [18].

## 2. Results

### 2.1. NFE2L2 and STAT3 Cooperate in Sustaining Cell Survival, MTOR, MAPK3/1 Activation, and Protein Synthesis in PEL Cells

**Figure 1:**
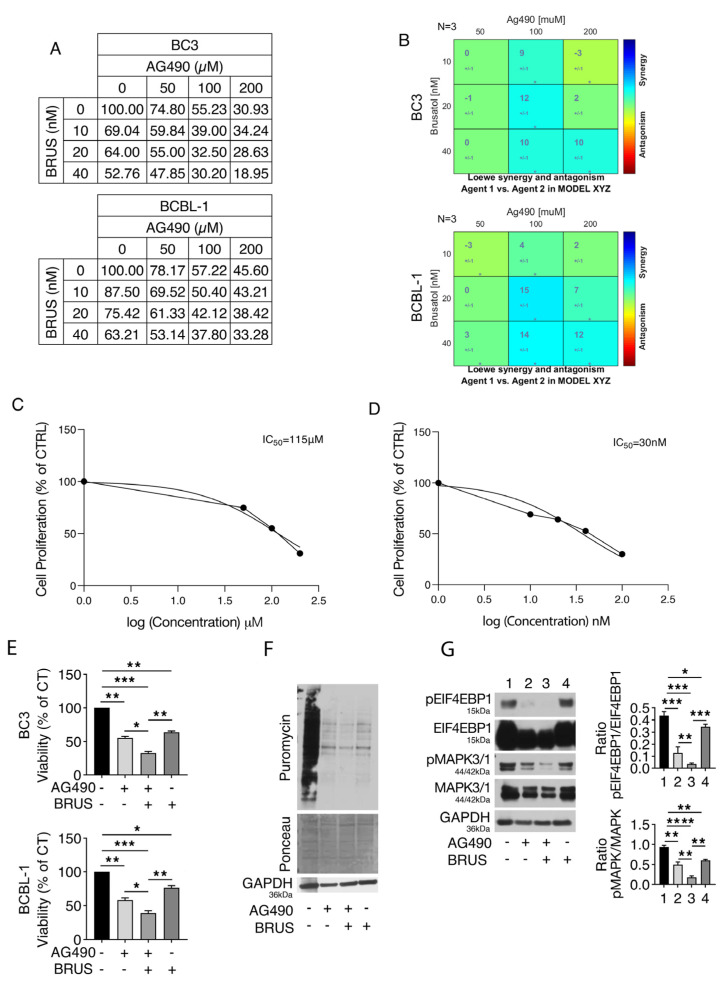
Concomitant NFE2L2 and STAT3 inhibition reduces proliferation, survival, MTOR and MAPK3/1 activation, and protein synthesis in PEL cells. BC3 and BCBL-1 cell lines were treated for 24 h singly or in combinations with different doses of AG490 (50–100–200 µM) and brusatol (BRUS, 10–20–40 nM). (**A**) Cell proliferation was evaluated by performing an MTT assay. Absorbance of treated cells was calculated as a percentage relative to untreated control cells. (**B**) Analysis and visualization of AG490 (50–100–200 µM) and brusatol (10–20–40 nM) combinations with Combenefit. *p*-value versus control: * <0.05. BC3 cell line concentration–response curve and IC_50_ value for AG490 (**C**) and brusatol (**D**) after treatment with various doses of AG490 (50–100–200 μM) and brusatol (10–20–40–100 nM) for 24 h calculated using Graphpad Prism^®^ software (version 9; Graphpad Software Inc., La Jolla, CA, USA). (**E**) Cell survival was estimated by trypan blue exclusion assay after treatment with AG490 (100 μM), brusatol (BRUS) (20 nM), or a combination of both for 24 h. The histograms represent the mean of the percentage of cell viability relative to the control plus S.D. *p*-value: * <0.05; ** <0.01; and *** <0.001. Puromycin (10 μg/mL) was added to BC3 cells in the last 30 min of AG490 (100 µM) and/or brusatol (BRUS) (20 nM) treatment, and (**F**) nascent protein level was evaluated by SUnSET assay. Ponceau staining and GAPDH were used to confirm equal loading, and one representative experiment is shown. BC3 cells were treated with AG490 (100 μM), brusatol (BRUS) (20 nM), or a combination of both for 24 h, and (**G**) protein expression level of pEIF4EBP1 (Thr37/46), EIF4EBP1, pMAPK3/1 (pMAPK), and MAPK3/1 (MAPK) was evaluated by Western blot analysis. GAPDH was used as a loading control, and one representative experiment is shown. The histograms represent the densitometric analysis of pEIF4EBP1 (Thr37/46)/EIF4EBP1 and pMAPK/MAPK. Data are represented as the mean plus S.D. *p*-value: * <0.05; ** <0.01; *** <0.001; and **** <0.0001.

To evaluate the impact of NFE2L2 and STAT3 concomitant inhibition on PEL cell proliferation, we treated BC3 and BCBL-1 cells with different doses of AG490 STAT3 inhibitor (50–100–200 μM), brusatol (10–20–40 nM), known to target NFE2L2 pathway or combination of both, and performed an MTT assay. We found that both drugs impaired cell proliferation and that they induced a synergic effect when used in combination (100 μM AG490/20 nM brusatol), compared to the control (DMSO), based on KERN index that yielded a value > 1 and on Loewe synergy models (Figure 1A,B), using the free software tool Combenefit (2.02 version) [19], and with the IC_50_ value of 115 μM and 30 nM for AG490 and brusatol, respectively (Figure 1C,D).

The synergic effect was also observed on cell survival when these drugs were used in combination at the same concentrations (100μM AG490/20 nM brusatol), as evaluated by a trypan blue assay (Figure 1E). Interestingly, the cytotoxic effect induced by AG490/brusatol correlated with a stronger reduction in protein synthesis compared to the AG490 or brusatol single treatments (Figure 1F) and a stronger inhibition of the two main molecular pathways sustaining protein synthesis, namely the MTOR/EIF4EBP1 axis and MAPK3/1 (Figure 1G) [20,21]. Altogether, these findings suggest that AG490/brusatol used in combination induced a stronger cytotoxic effect against PEL cells compared to the single treatments.

### 2.2. NFE2L2 and STAT3 Sustain Each Other and Converge in the Regulation of Several Targets

**Figure 2:**
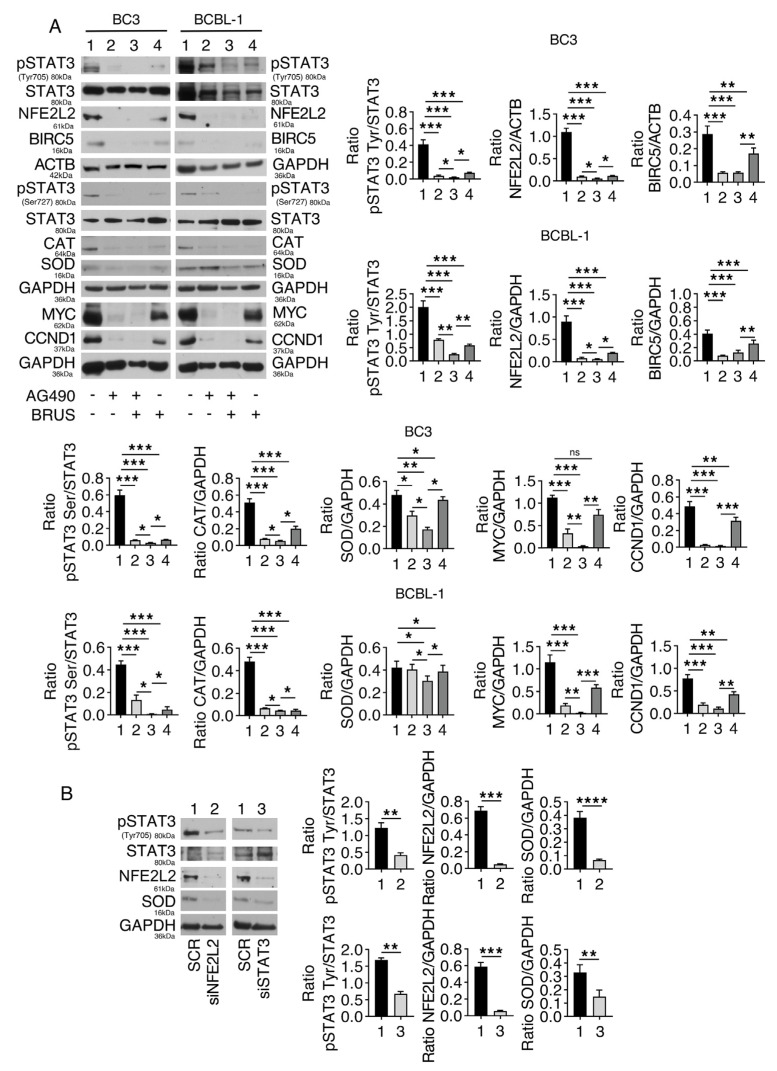
Molecules regulated by STAT3 and NFE2L2 signaling in PEL cells. (**A**) BC3 and BCBL-1 cells were treated with AG490 (100 μM), brusatol (BRUS) (20 nM), or a combination of both for 24 h, and protein expression level of pSTAT3 on Tyr705, pSTAT3 on Ser727, STAT3, catalase (CAT), superoxide dismutase 1 (SOD), NFE2L2, MYC, CCND1, and BIRC5 was evaluated by Western blot analysis. (**B**) NFE2L2 or STAT3 were silenced in BC3 cells, and the protein expression level of pSTAT3 on Tyr705, STAT3, NFE2L2, and SOD was evaluated by Western blot analysis. ACTB or GAPDH was used as a loading control, and one representative experiment is shown. The histograms represent the densitometric analysis of the ratio of specific proteins and the appropriate control. Data are represented as the mean plus S.D. *p*-value: * <0.05; ** <0.01; *** <0.001; and **** <0.0001.

NFE2L2 is the main transcription factor devolved at protecting cells from oxidative stress by transcribing phase I and II antioxidant enzymes [22]. STAT3 also plays an important role in cancer cell survival, including PEL cells [16,23], by regulating the expression of molecules such as BIRC5 [24]. Here, we asked whether NFE2L2 and STAT3 could cross-talk and cooperate in promoting the survival of PEL cells. We found that AG490, besides reducing STAT3 phosphorylation on tyrosine residue 705 (Tyr705) and serine residue 727 (Ser727), reduced the NFE2L2 expression level and that of its target CAT (Figure 2A). On the other hand, brusatol, which inhibits NFE2L2, also reduced STAT3 activation (Figure 2A) and downregulated MYC, CCND1, and BIRC5, considered to be STAT3 targets (Figure 2A). Moreover, the concomitant inhibition of NFE2L2 and STAT3 induced a stronger downregulation of MYC and CCND1 (Figure 2A), suggesting that they converged in the regulation of several targets in both BC3 and BCBL-1 cell lines. The reciprocal positive regulation between these transcription factors was also shown by performing silencing experiments showing that the knocking down of NFE2L2 inhibited STAT3 and that the silencing of STAT3 inhibited NFE2L2 (Figure 2B), confirming that these molecules were able to sustain each other’s activation.

### 2.3. HSPs and p62/SQSTM1 Are Involved in the NFE2L2 and STAT3 Interplay

**Figure 3:**
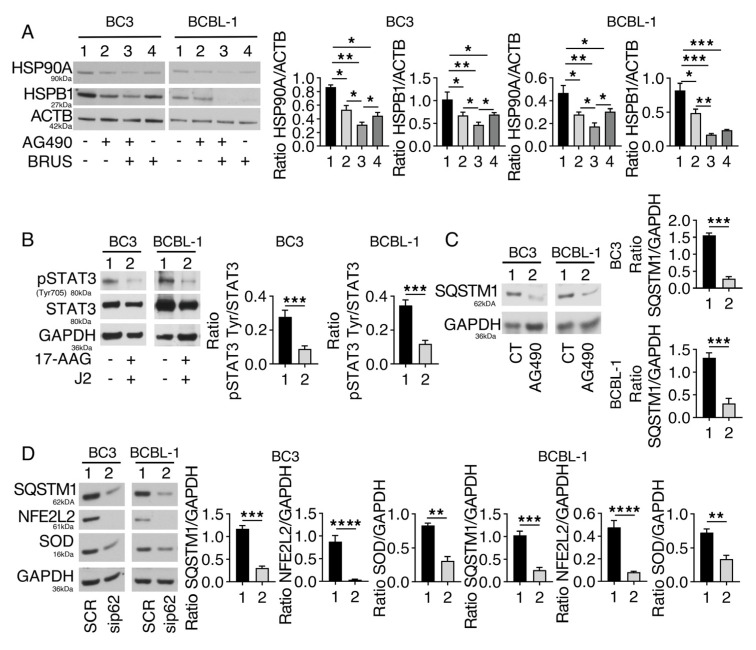
HSPs and p62/SQSTM1 contribute to the positive feedback loop between NFE2L2 and STAT3. PEL cell lines were treated with AG490 (100 μM), brusatol (BRUS) (20 nM), or a combination of both for 24 h, and (**A**) protein expression level of HSP90A and HSPB1 was evaluated by Western blot analysis. ACTB was used as a loading control, and one representative experiment is shown. The histograms represent the densitometric analysis of the ratio of specific protein/ACTB. Data are represented as the mean plus S.D. *p*-value: * <0.05; ** <0.01; and *** <0.001. BC3 and BCBL-1 cells were treated with inHSPB1 (J2) (10 µM) and inHSP90A (17-AAG) (0.1 µM) for 24 h, and (**B**) protein expression levels of pSTAT3 on Tyr705 and STAT3 was evaluated by Western blot analysis. GAPDH or ACTB was used as a loading control, and one representative experiment is shown. The histograms represent the densitometric analysis of pSTAT3 (Tyr705)/STAT3. Data are represented as the mean plus S.D. *p*-value: *** <0.001. BC3 and BCBL-1 cells were treated with AG490 (100 μM) for 24 h, and (**C**) protein expression level of p62/SQSTM1 (p62) was evaluated by Western blot analysis. GAPDH was used as a loading control, and one representative experiment is shown. The histograms represent the densitometric analysis of the ratio of p62/GAPDH. Data are represented as the mean plus S.D. *p*-value: *** <0.001. p62/SQSTM1 was silenced in BC3 and BCBL-1 cells, and (**D**) protein expression level of p62/SQSTM1 (p62), NFE2L2, and SOD was evaluated by Western blot analysis. GAPDH was used as a loading control, and one representative experiment is shown. The histograms represent the densitometric analysis of the ratio of specific protein/GAPDH. Data are represented as the mean plus S.D. *p*-value: ** <0.01; *** <0.001; and **** <0.0001.

We then investigated the possible mechanism/s involved in NFE2L2/STAT3 interplay. NFE2L2 is known to contribute to the transcription of HSPs, molecules reported to chaperone kinases that maintain STAT3 in an activated state [4]. Therefore, we evaluated the expression of HSPs such as HSP90A and HSPB1 in PEL cells undergoing brusatol treatment and found that both chaperones were downregulated (Figure 3A). To demonstrate that HSP90A and HSPB1 were involved in sustaining STAT3 activation in this setting, we pharmacologically inhibited both HSPs and found that such treatment de-phosphorylated STAT3 (Figure 3B), suggesting that the HSPs reduced expression was involved in STAT3 de-phosphorylation mediated by brusatol. To investigate the possible mechanisms through which STAT3 could sustain NFE2L2, we evaluated the role of p62/SQSTM1. Indeed, AG490, by inducing autophagy, was previously found to reduce its expression level in PEL cells [16], and p62/SQSTM1 is known to stabilize NFE2L2. In this study, we first confirmed that the expression level of p62/SQSTM1 was reduced by AG490 treatment (Figure 3C), and then, to demonstrate that p62/SQSTM1 could stabilize NFE2L2, we silenced it by specific small interference RNA (siRNA). The results shown in Figure 3D indicate that p62/SQSTM1 knockdown reduced NFE2L2 expression and activity, suggesting that its downregulation by AG490 could contribute to inhibiting NFE2L2 in PEL cells. Finally, we found that also AG490 reduced HSP90A and HSPB1, in agreement with previous findings [7], and that the combination of AG490/brusatol more efficiently reduced the expression level of these HSPs (Figure 3A). These results suggest that inhibition of NRF2 mediated by AG490 could contribute to the reduction of p62/SQSTM1 and HSPs.

### 2.4. STAT3 and NFE2L2 Concomitant Inhibition More Efficiently Reduces ATM and Molecules Belonging to Both HR and NHEJ DNA Repair Pathways by Downregulating HSPB1 and HSP90A

**Figure 4:**
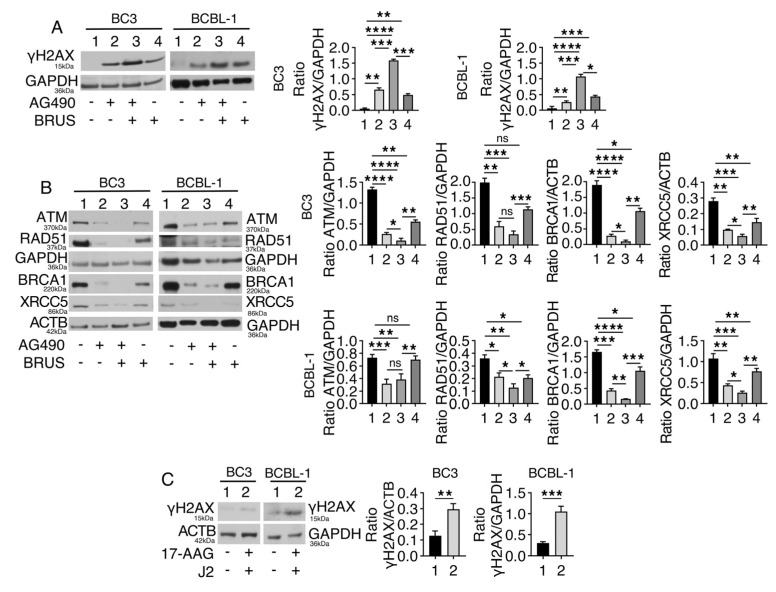
Concomitant NFE2L2 and STAT3 inhibition induces strong DNA damage in PEL cells, which correlates with the downregulation of molecules involved in DNA repair. PEL cell lines were treated with AG490 (100 μM), brusatol (BRUS) (20 nM), or a combination of both for 24 h, and (**A**) protein expression level of γH2AX was evaluated by Western blot analysis. GAPDH was used as a loading control, and one representative experiment is shown. The histograms represent the densitometric analysis of the ratio of γH2AX/GAPDH. Data are represented as the mean plus S.D. *p*-value: * <0.05; ** <0.01; *** <0.001; and **** <0.0001. (**B**) Protein expression level of ATM, BRCA1, RAD51, and XRCC5 was evaluated in PEL cells treated with AG490 (100 μM), brusatol (BRUS) (20 nM), or a combination by Western blot analysis. GAPDH was used as a loading control, and one representative experiment is shown. The histograms represent the densitometric analysis of the ratio of specific protein/GAPDH. Data are represented as the mean plus S.D. *p*-value: not significative (ns); * <0.05; ** <0.01; *** <0.001; and **** <0.0001. BC3 and BCBL-1 cells were treated with inHSPB1 (J2) (10 µM) and inHSP90A (17-AAG) (0.1 µM) for 24 h, and (**C**) protein expression level of γH2AX was evaluated by Western blot analysis. ACTB or GAPDH was used as a loading control, and one representative experiment is shown. The histograms represent the densitometric analysis of the ratio of γH2AX/GAPDH. Data are represented as the mean plus S.D. *p*-value: ** <0.01; *** <0.001.

In correlation with the higher cytotoxic effect induced by AG490/brusatol combination, we then found that stronger DNA damage was induced in PEL cells, as indicated by the increase in γH2AX expression level (Figure 4A). To investigate whether this effect could correlate with the impairment of DDR, we evaluated the expression of ataxia telangiectasia mutated kinase (ATM), a kinase that triggers DDR signaling cascade in response to a DNA double-strand break, breast cancer type 1 susceptibility protein (BRCA1), and RAD51, molecules involved in HR, and XRCC5, belonging to NHEJ DNA repair pathways [25]. As shown in Figure 4B, AG490, brusatol, and even more, the combination of both reduced the expression of all these DDR molecules, suggesting that the DNA damage induced by the AG490/brusatol combination correlated with a stronger impairment of DDR. As AG490/brusatol downregulated several DDR molecules and reduced the expression of HSP90A and HSPB1, of which these DDR proteins are clients [7], we investigated whether the inhibition of HSP90A and HSPB1 could also induce DNA damage in PEL cells. As shown in Figure 4C, we found that this was the case, as the expression of γH2AX increased following the concomitant inhibition of HSP90A and HSPB1.

## 3. Discussion

**Figure 5:**
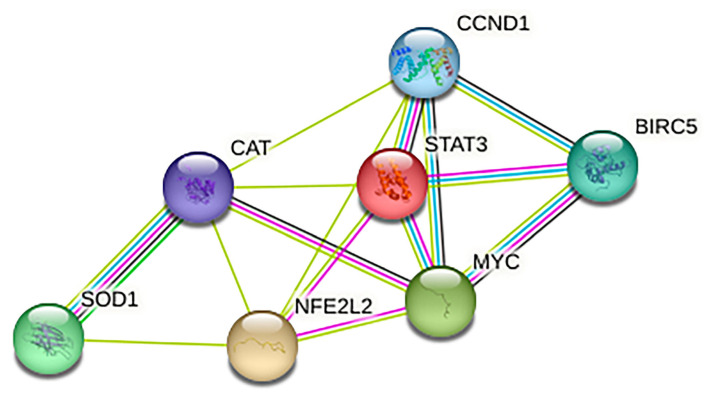
STAT3/NFE2L2 interactome as identified by using STRING-query.

Targeting STAT3 represents a promising strategy for the treatment of aggressive cancers displaying a constitutive activation of this pathway, including PEL, which is very aggressive B cell lymphoma KSHV-associated [23]. STAT3 is indeed essential for the expression of pro-survival molecules, and it is interconnected with the production of IL-6, a cytokine that has been shown to contribute to PEL cell growth/proliferation [26]. However, a cross-talk between oncogenic pathways may occur in cancer cells, and the targeting of one pathway may result in the activation of others, leading to chemoresistance [27]. One possibility that may help to overcome such a problem is the concomitant inhibition of several oncogenic pathways, e.g., by using multitargeting drugs such as natural compounds, as we have shown for quercetin [28], or by combining the inhibition of different pathways. In this study, we found that the concomitant inhibition of STAT3 and NFE2L2 exerted a stronger cytotoxic effect against PEL compared to the inhibition of each of these molecules. Indeed, by targeting STAT3 and NFE2L2, the activation of MTOR and MAPK3/1 were more efficiently inhibited, and accordingly, protein synthesis was more strongly reduced. Indeed, MTOR and MAPK3/1 pathways, among other important cellular processes, can positively regulate protein synthesis, which is required to sustain cancer cell survival and proliferation [20]. Moreover, we found that NFE2L2 and STAT3 converged in the regulation of several proteins. Indeed, besides its own targets, NFE2L2 inhibition reduced those considered to be STAT3 targets, and on the other way around, STAT3 inhibition reduced NFE2L2 targets. Accordingly, by performing an online STRING-query on STAT3, NFE2L2, and their targets [29], we obtained the STAT3/NFE2L2 interactome, which shows that these transcription factors may share the control of molecules considered specific to each of them (Figure 5) [30]. Notably, HSP90A and HSPB1 were involved in STAT3/NFE2L2 interplay, as their expression level was downregulated by STAT3, by NFE2L2, and more efficiently by the concomitant inhibition of both transcription factors. As we have previously shown, HSP reduction contributed to inducing strong DNA damage in PEL cells, given that several DDR molecules, including ATM, BRCA1, RAD51, and XRCC5, are clients of HSPs [18] and, therefore, they were strongly reduced following AG490/brusatol treatment. The reduction of HSPs has been reported to lead to de-phosphorylation of important pathways, including STAT3, as kinases responsible for STAT3 activation are also among the numerous HSPs clients [4]. This finding was confirmed in the present study, in which the inhibition of both HSPB1 and HSP90A strongly reduced the phosphorylation of STAT3. Interestingly, HSP inhibition also resulted in the inhibition of NFE2L2 activity, which may suggest that its stabilization could be mediated by these HSPs. However, the reduction of p62/SQSTM1 by AG490 contributed to NFE2L2 inhibition, as demonstrated by p62/SQSTM1 silencing and according to the knowledge that NFE2L2 can be stabilized by p62/SQSTM1 [14]. Previous studies, including our own, have shown that targeting NFE2L2 could be a promising strategy to reduce cell survival of several cancer types and to counteract chemoresistance when used in combination with other drugs [31,32]. However, the NFE2L2 and STAT3 cross-talk and the impact of their concomitant inhibition on cell survival have not been completely elucidated yet, particularly in the context of PEL cells. Regarding this lymphoma, it has been shown that NFE2L2 could control the KSHV latent/lytic switch [33,34] and that its activation by dimethyl fumarate could induce a cytotoxic effect against PEL cells, as we have previously demonstrated [35]. Therefore, based on the present and previous studies, it seems that proper activation of NFE2L2 is required to sustain lymphoma cell survival and regulate KSHV lytic cycle activation. The same has been reported to occur for ROS, strictly controlled by NFE2L2, whose level should be not too high and also not too low, in order to maintain activated oncogenic pro-survival pathways, sustain cell survival [36,37], and allow viral replication as well [34].

In conclusion, this study shows for the first time that NFE2L2 and STAT3 engage a positive regulatory circuit that promotes PEL cell survival. These transcription factors cooperate to control the activation of pathways such as MTOR and MAPK3/1, protein synthesis, and share the positive regulation of antioxidant enzymes such as CAT and of molecules such as MYC, BIRC5, and CCND1 that promote cell survival or proliferation. Notably, NFE2L2 and STAT3 together promote the expression of HSPs such as HSP90A and HSPB1, from which the stability of DDR molecules and DNA damage repair strongly depend, which further indicates their importance in the control of PEL cell homeostasis.

## 4. Materials and Methods

### 4.1. Cell Cultures and Treatments

Human B cell lines derived from KSHV-positive PEL cell lines, BC3, and BCBL-1 (kindly supplied by Prof. P. Monini, National AIDS center, Istituto Superiore di Sanità, Rome, Italy) were grown in RPMI 1640 medium (Sigma-Aldrich, Burlington, MA, USA) supplemented with 10% fetal bovine serum (FBS) (Sigma-Aldrich, Burlington, MA, USA), L-glutamine (2 mM) (Aurogene, Rome, Italy), and streptomycin/penicillin (100 μg/mL) (Aurogene, Rome, Italy) (complete medium) at 37 °C in a 5% CO_2_ humified setting. Cells were seeded into 6-well plates at a density of 4 × 10^5^ per well in a final volume of 2 mL in complete medium and were treated for 24 h (h) singly or in combinations with the STAT3 inhibitor tyrphostin AG490 (50–100–200 µM) (Calbiochem, San Diego, CA, USA; 658411) and the NFE2L2 inhibitor brusatol (10–20–40 nM) (Sigma Aldrich, St. Louis, MO, USA; 1868). In some experiments, to evaluate the role of HSP90A and HSPB1 in the mechanism/s to which NFE2L2 could sustain STAT3 activity and vice versa, PEL cells were treated for 24 h with inhibitors of HSPB1 and HSP90A, respectively, J2 (10 µM) (MedChemExpress, Monmouth Junction, NJ, USA; HY-124653), 17-AAG (0.1 µM) (Selleckem, Planegg, Germany; S1141). All the drugs were dissolved in DMSO, and the control cells were supplemented with DMSO in the same amount used for the other samples.

### 4.2. MTT Assay

Cell proliferation was determined by 3-(4,5-dimethylthiazol-2-yl)-2,5-diphenyl tetrazolium bromide (MTT, Sigma-Aldrich, St. Louis, MO, USA) assay.

MTT assay measures cell proliferation based on the enzymatic conversion of tetrazolium salt (MTT) into its insoluble formazan by dehydrogenases resident in the mitochondria of living cells. The conversion is evidenced by the color change from yellow to purple.

PEL cells were plated in 96-well plates at a density of 20 × 10^3^ cells/well in 100 μL of RPMI 1640 complete medium and treated with AG490 and/or brusatol for 24 h. After treatments, 10 µL of MTT reagent was added to each well, the plate was incubated for 4 h at 37 °C in a 5% CO_2_ incubator, and 100 μL of the solubilization buffer was added into each well. The plates were kept at 37 °C in a 5% CO_2_ incubator, and the following day the intensity of formazan staining was determined by measuring optical density at 560 nm wavelength with an Absorbance 96 reader (Byonoy GmbH, Hamburg, Germany). The absorbance of treated cells was plotted relative to control cells. The experiments were performed in triplicate and repeated three times.

Loewe synergy models were used to determine if the AG490/brusatol combination induced a synergistic cytotoxic by using the free software tool Combenefit (version 2.02).

The mean inhibition concentration (IC_50_) values of AG490 and brusatol were calculated using Graphpad Prism^®^ software (version 9; Graphpad Software Inc., La Jolla, CA, USA).

### 4.3. Cell Assay Viability

After AG490/brusatol (100 µM/20 nM) treatment, a trypan blue (Sigma-Aldrich, Burlington, MA, USA, 72571) dye exclusion assay was performed to determine the number of viable cells. Unstained cells (live cells) were counted by light microscopy using a Neubauer hemocytometer. The experiments were performed in triplicate and repeated at least three times.

### 4.4. Protein Synthesis Assay—Surface Sensing of Translation (SUnSET)

SUnSET assay is based on the use of puromycin that is an analog of aminoacyl tRNAs with a modified adenosine covalently linked to a tyrosine amino acid that, when incorporated into the nascent polypeptide chain, prevents the elongation. When used at low doses, puromycin incorporation in neosynthesized proteins directly reflects the rate of mRNA translation in vitro.

To evaluate protein synthesis, puromycin (10 μg/mL) was added to cells in the last 30 min (min) of AG490 (100 µM) and/or brusatol (20nM) experiments. After incubation, the cells were centrifuged at 1200 rpm (revolutions per minute) for 5 min at room temperature (RT), washed once in 1x PBS, lysed in RIPA buffer (150 mM NaCl, 1% NP-40, 50 mM Tris-HCl (pH 8), 0.5% deoxycholic acid, 0.1% SDS, protease, and phosphatase inhibitors) and centrifuged at 14,000 rpm for 45 min at 4 °C to remove cellular debris. Total protein concentration was measured by using Quick Start Bovine Serum Albumin (BSA) assay (Bio-Rad, Hercules, CA, USA), and 15 μg of protein was denatured in loading buffer by heating for 10 min at 70 °C and was subjected to electrophoresis on 4–12% NuPage Bis-Tris gels (Life Technologies, Carlsbad, CA, USA), according to the manufacturer’s instruction. The gels were transferred to nitrocellulose membranes (Bio-Rad, Hercules, CA, USA) for 45 min in tris-glycine buffer, and then the membranes were stained with Ponceau S staining solution (SERVA Electrophoresis GmbH, Heidelberg, Germany, 33427.01) to verify protein transfer. The membranes were washed and blocked in 1x PBS-0.1% Tween20 solution containing 2% of BSA (SERVA Electrophoresis GmbH, Heidelberg, Germany, 11946.02) for 1 h. Subsequently, membranes were incubated with mouse anti-puromycin antibody (1:25,000) (clone 12D10) (Sigma Aldrich, MABE343, Burlington, MA, USA) and developed using ECL Blotting Substrate (Advansta, San Jose, CA, USA).

### 4.5. Western Blot Analysis

After treatments, the cells were harvested, centrifuged at 1200 rpm for 5 min at RT, and cell pellet lysed in RIPA buffer (150 mM NaCl, 1% NP-40, 50 mM Tris-HCl (pH 8), 0.5% deoxycholic acid, 0.1% SDS, protease, and phosphatase inhibitors). The protein concentration was measured by using Quick Start Bovine Serum Albumin (BSA) assay (Bio-Rad, Hercules, CA, USA), and 15 μg of protein was denatured in loading buffer by heating for 10 min at 70 °C and subjected to electrophoresis on 4–12% NuPage Bis-Tris gels (Life Technologies, Carlsbad, CA, USA) according to the manufacturer’s instruction. The gels were transferred to nitrocellulose membranes (Bio-Rad, Hercules, CA, USA) for 45 min in tris-glycine buffer, and then the membranes were stained with Ponceau S staining solution (SERVA Electrophoresis GmbH, Heidelberg, Germany, 33427.01) to verify protein transfer. The membranes were washed and blocked in 1× PBS-0.1% Tween20 solution containing 2% of BSA (SERVA Electrophoresis GmbH, Heidelberg, Germany, 11946.02) for 1h at RT, incubated with specific antibodies, and developed using ECL Blotting Substrate (Advansta, San Jose, CA, USA). The antibodies used are listed in Table S1 in Supplementary Section.

### 4.6. Knockdown of NFE2L2, STAT3 and p62/SQSTM1 by Small Interfering RNA (siRNA)

NFE2L2, STAT3, and p62/SQSTM1 knockdown were performed by specific siRNA using INTERFERin transfection reagent (Polypolus Transfection, Illkirch-Graffenstaden, France, 409-50) according to the manufacturer’s instructions. Briefly, PEL cells were seeded into 12-well plates at a density of 4 × 10^5^ cells per well and transfected with 1.8 pmol of NFE2L2 (NRF2)-siRNA (siNRF2, Santa Cruz Biotechnology Inc., Dallas, TX, USA, sc-29226), STAT3-siRNA (siSTAT3, Santa Cruz Biotechnology Inc., Dallas, TX, USA, sc-29493), and p62/SQSTM1-siRNA (sip62/SQSTM1, Santa Cruz Biotechnology Inc., Dallas, TX, USA, sc-29679). Control siRNA-A (Santa Cruz Biotechnology, Dallas, TX, USA, sc-37007) was used as a scrambled control (SCR). The cells were collected after 48 h of transfection for subsequent analysis.

### 4.7. Densitometric Analysis

The quantification of protein bands was performed by densitometric analysis using the Image J software (1.47 version, NIH, Bethesda, MD, USA), which was downloaded from the NIH website (http://imagej.nih.gov, accessed on 10 February 2022).

### 4.8. Statistical Analysis

Results are represented by the mean plus standard deviation (S.D.) of at least three independent experiments, and statistical analyses were performed with Graphpad Prism^®^ software (version 9; Graphpad Software Inc., La Jolla, CA, USA). Student’s *t*-test or a nonparametric one-way ANOVA test was used to demonstrate statistical significance. The difference was considered statistically significant when the *p*-value was: * <0.05; ** <0.01; *** <0.001; and **** <0.0001. Not significative (ns).

## Data Availability

Data are available upon reasonable request from the corresponding author.

## Supplementary Materials

The following supporting information can be downloaded at: https://www.mdpi.com/article/10.3390/ijms241411598/s1.
