# Supplementary material for: NFE2L2 and STAT3 Converge on Common Targets to Promote Survival of Primary Lymphoma Cells
Source: Int J Mol Sci. 2023 Jul 18;24(14):11598. doi: 10.3390/ijms241411598 (PMC10380615; doi:10.3390/ijms241411598)
Supplement: Supplementary file 1 [file ijms-24-11598-s001.zip › ijms-2502753-supplementary.pdf]

Supplementary Materials

Supplementary Table S1. List of Antibodies used to identify specific proteins in western blot analyses are listed below:

**Antibody**	**Isotype**	**Species**	**Dilution ratio**	**Company**	**Catalog #**
pEIF4EBP1 (Thr37/46)	Policlonal	Rabbit	1:500	Cell Signaling	2855
EIF4EBP1	Policlonal	Rabbit	1:500	Proteintech	60246-1-Ig
pMAPK3/1	Monoclonal	Mouse	1:500	Santa Cruz Biotechnology	sc-7383
MAPK3	Policlonal	Rabbit	1:500	Santa Cruz Biotechnology	sc-93
MAPK1	Policlonal	Rabbit	1:500	Santa Cruz Biotechnology	sc-154
pSTAT3 Tyr705	Policlonal	Rabbit	1:500	Cell Signaling	9145
pSTAT3 Ser727	Policlonal	Rabbit	1:500	Cell Signaling	9134
STAT3	Monoclonal	Mouse	1:500	Santa Cruz Biotechnology	sc-482
CAT	Monoclonal	Mouse	1:200	Santa Cruz Biotechnology	sc-271803
SOD	Policlonal	Rabbit	1:500	Proteintech	10269-1-AP
NFE2L2	Monoclonal	Mouse	1:100	Santa Cruz Biotechnology	sc-365949
MYC	Policlonal	Rabbit	1:500	Proteintech	10828-1-AP
CCND1	Monoclonal	Mouse	1:100	Santa Cruz Biotechnology	sc-8396
BIRC5	Policlonal	Rabbit	1:1000	Proteintech	10508-1-AP
HSP90A	Policlonal	Rabbit	1:5000	Proteintech	13171-1-AP
HSPB1	Policlonal	Rabbit	1:5000	Proteintech	18284-1-AP
p62/SQSTM1	Policlonal	Rabbit	1:1000	Cell Signaling	5114T
γH2AX (Ser 139)	Monoclonal	Mouse	1:100	Santa Cruz Biotechnology	sc-517348
ATM	Monoclonal	Mouse	1:100	Santa Cruz Biotechnology	sc-135663
BRCA1	Monoclonal	Mouse	1:1000	EMD Millipore	OP92
RAD51	Monoclonal	Mouse	1:100	Santa Cruz Biotechnology	sc-377467
XRCC5	Monoclonal	Mouse	1:100	Santa Cruz Biotechnology	sc-5280
GAPDH	Monoclonal	Mouse	1:10000	Santa Cruz Biotechnology	sc-137179
ACTB	Monoclonal	Mouse	1:10000	Sigma Aldrich	A5316

All the primary and secondary antibodies were diluted in 1x PBS-0.1% Tween20 solution containing 2% of BSA (SERVA Electrophoresis GmbH, Heidelberg, Germany).
